# Transparent Conducting Films Based on Carbon Nanotubes: Rational Design toward the Theoretical Limit

**DOI:** 10.1002/advs.202201673

**Published:** 2022-06-16

**Authors:** Daniil A. Ilatovskii, Evgeniia P. Gilshtein, Olga E. Glukhova, Albert G. Nasibulin

**Affiliations:** ^1^ Skolkovo Institute of Science and Technology Nobel Str. 3 Moscow 143026 Russian Federation; ^2^ Empa‐Swiss Federal Laboratories for Materials Science and Technology Überlandstrasse 129 Dübendorf 8600 Switzerland; ^3^ Saratov State University Astrakhanskaya Str. 83 Saratov 410012 Russian Federation; ^4^ I.M. Sechenov First Moscow State Medical University Bolshaya Pirogovskaya Str. 2–4 Moscow 119991 Russian Federation; ^5^ Aalto University Espoo FI‐00076 Finland

**Keywords:** carbon nanotubes, chemical vapor deposition (CVD), optoelectronics, transparent conductors

## Abstract

Electrically conductive thin‐film materials possessing high transparency are essential components for many optoelectronic devices. The advancement in the transparent conductor applications requires a replacement of indium tin oxide (ITO), one of the key materials in electronics. ITO and other transparent conductive metal oxides have several drawbacks, including poor flexibility, high refractive index and haze, limited chemical stability, and depleted raw material supply. Single‐walled carbon nanotubes (SWCNTs) are a promising alternative for transparent conducting films (TCFs) because of their unique and excellent chemical and physical properties. Here, the latest achievements in the optoelectronic performance of TCFs based on SWCNTs are analyzed. Various approaches to evaluate the performance of transparent electrodes are briefly reviewed. A roadmap for further research and development of the transparent conductors using “rational design,” which breaks the deadlock for obtaining the TCFs with a performance close to the theoretical limit, is also described.

## Graphical abstract

The field of optoelectronics is in dire need of novel materials combining both high transparency and electrical conductivity. This review is dedicated to the most promising material for transparent conductors – single‐walled carbon nanotubes. The authors overview here outstanding achievements, introduce the universal indicator for comparison of all transparent conducors, and describe the roadmap for further development of the topic.

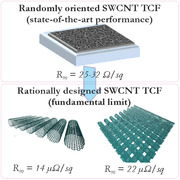

## 1 Introduction

During the last two decades, transparent conducting films have become a rapidly developing field of materials science. They are key elements in modern electronic devices such as solar cells,^[^ ^1^, ^2^, ^3^, ^4^, ^5^, ^6^ ^]^ organic light‐emitting diodes,^[^ ^7^, ^8^, ^9^, ^10^, ^11^ ^]^ liquid crystal displays,^[^ ^12^, ^13^, ^14^ ^]^ touch screens,^[^ ^15^, ^16^, ^17^ ^]^ and wearable devices.^[^ ^18^, ^19^, ^20^ ^]^ Several materials are used to produce transparent electrodes: metal oxides, conducting polymers, metallic thin films, and micro‐ or nanostructured surfaces, films based on carbon nanomaterials and their composites.^[^ ^21^, ^22^ ^]^ Nowadays, nearly three‐quarters of electronic devices using transparent conductors are based on indium tin oxide (ITO). Even though ITO exhibits excellent optoelectronic performance, the market is looking for an adequate replacement. New trends in electronics require a flexible form factor, which ITO cannot provide because of its fragile nature. Furthermore, ITO has a high refractive index and haze, spectrally nonuniform optical transmission, poor chemical stability, and depleted raw material supply. Therefore, to further advance the field of transparent conductors, new technologies and materials have to be developed and explored, eliminating the disadvantages.^[^ ^23^, ^24^, ^25^ ^]^ Unfortunately, other transparent conducting metal oxides, such as ZnO doped by Ga or Al;^[^ ^26^, ^27^ ^]^ TiO_2_ doped by Nb, Ta, or W;^[^ ^28^ ^]^ or SnO_2_ doped by Sb, Nb, Ta, or F^[^ ^29^, ^30^, ^31^, ^32^ ^]^ cannot broaden the TCF application range due to similar limitations.

The conducting polymers, such as PEDOT:PSS (poly(3,4‐ethylene dioxythiophene) [PEDOT] doped with poly(styrene sulfonate) [PSS]^[^ ^33^, ^34^ ^]^), allow achieving transmittance values close to that of ITO, additionally overcoming the mechanical fragility problem. The conducting polymers are of limited conductivity and usually rapidly degrade,^[^ ^34^ ^]^ which seriously constrains their applicability in transparent electronics.

Metals represent another potential alternative for transparent electrodes. All metal‐based transparent conductors can be classified into three groups: thin films,^[^ ^35^, ^36^, ^37^ ^]^ microgrids,^[^ ^14^, ^38^, ^39^ ^]^ and nanowires.^[^ ^40^ ^]^ The first one is based on the fabrication of a continuous few nanometer thick film with low light absorption. The decrease in the film thickness results in the boundary scattering of electrons, which hinders the electrical conductivity, however, due to high transmittance allows to achieve good optoelectronic properties. The best results were obtained for Ca/Ag blend thin film and Al‐doped silver film correspondingly with sheet resistances of *R*_s_ = 27 Ω sq^−1^ at the transmittance of 93%^[^ ^41^ ^]^ and *R*_s_ = 20 Ω sq^−1^ at 92.4%.^[^ ^42^ ^]^ In general, the fabrication of metallic films with simultaneously high transmittance and conductance is a technologically challenging task.^[^ ^43^ ^]^ Pure metal films possess weak adhesion to substrates and require an additional adhesive layer.^[^ ^44^ ^]^ In addition to an energy‐consuming vacuum metal deposition, this method requires several pre‐ and post‐treatment steps, making it expensive for industrial purposes. The second group of metal‐based transparent conductors can be realized by a simple imprinting method in the form of micrometer‐scale patterns,^[^ ^45^ ^]^ similar to microstructured polymers^[^ ^46^ ^]^ or inorganic oxides^[^ ^47^ ^]^ prepared by the soft lithography technique. This approach allows achieving overall high transmittance values of the conductive films with the nontransparent thin metallic grids.^[^ ^48^, ^49^ ^]^ As the fabrication process of microgrids possesses a high level of nonuniformity, it does not offer a strategic improvement over the standard uniform ITO film fabrication. Furthermore, the structural periodicity of the grid lines might restrict applications because of the optical diffraction phenomenon. The third group of metal TCFs is based on networks of randomly oriented 1D metal nanowires with a diameter of less than 100 nm and a length of several micrometers.^[^ ^17^, ^49^, ^50^ ^]^ Currently, this group of TCFs is expected to be a promising alternative to ITO. However, despite the high conductivity of this and other metal‐based TCFs, their exploitation is limited by weak adhesion to the substrate, high surface roughness, fragility, and infeasibility to get films with uniform electrical field.^[^ ^21^, ^51^ ^]^

Another potential alternative for transparent electrodes is carbon nanomaterials, since they possess high conductivity. Graphene is a 2D carbon allotrope comprising a monoatomic layer in a hexagonal lattice with an optical transmittance of 97.7%^[^ ^52^ ^]^ and sheet resistance theoretically predicted to be as low as 30 Ω sq^−1^.^[^ ^53^ ^]^ Reported experimental results are promising: Bae et al.^[^ ^54^ ^]^ demonstrated the technique for the production of graphene films with the sheet resistance of *R*_s_ = 125 Ω sq^−1^ with the transmittance of 97.4% and 30 Ω sq^−1^ for 90% of layer‐by‐layer stacked four‐layer graphene film. The formation of small graphene grains during the synthesis and subsequent transfer result in an increase in the film resistance due to the appearance of electric junctions^[^ ^55^, ^56^, ^57^ ^]^ and defects.^[^ ^58^ ^]^ The current state of the graphene TCF research has been thoroughly reviewed in the literature.^[^ ^5^, ^59^, ^60^, ^61^, ^62^, ^63^ ^]^

Single‐walled carbon nanotubes (SWCNTs) are 1D objects, which can be gathered as a thin network that exhibits high electrical conductivity and optical transparency. When dealing with SWCNT films, their properties can be adjusted by individual nanotubes (changing their length, diameter, doping level, chirality, etc.) and by their structure (varying thickness, density, alignment, patterning as well as composing the tubes with other materials). Compared to other potential TCF materials, SWCNT films enable an easier fabrication process and provide a flexible and stretchable platform for the development of future electronics. A few surveys have been recently published^[^ ^60^, ^64^, ^65^, ^66^, ^67^, ^68^ ^]^ describing the current state of the SWCNT TCF topic.

Here, we critically review the field of TCFs based on SWCNTs and analyze various methods of the TCF performance evaluation. Then, we outline and compare the key principles of SWCNT‐based TCF fabrication processes. We describe a rational design approach to fabricate transparent conductors, analyzing the global roadmap for future developement. Finally, we reveal the ultimate target for the carbon nanotube TCF development, i.e., an ideal network of periodically oriented defect‐free SWCNTs, revealing the fundamental limit of their optoelectrical performance. We also characterize stretchable TCFs to point out the perspective development of the conductors beyond rigid transparent materials.

## 2 Quality Factors for TCFs

Depending on the demand, method, and material, TCFs are usually produced with different thicknesses and, therefore, transparency and conductivity. To compare the performance of different TCF materials, we need to introduce a figure of merit (FoM) or quality factor, which is calculated based on film properties such as transmittance (*T*) and sheet resistance (*R*_s_) and valid for an arbitrary thickness. Transmittance is the intensity ratio of the incident (*I*_0_) and transmitted radiation (*I*):

(1) $$T=\frac{I}{I_{0}}=e^{-\alpha t}$$

where *t* is the thickness of the material (cm), and *α* is the coefficient of optical absorption (cm^−1^). The sheet resistance of the material at direct current (dc) is defined as:

(2) $$R_{s}=\frac{1}{\sigma_{\mathrm{dc}}\cdot t}$$

where *σ*_dc_ is the electrical conductivity (Ω^−1^ cm^−1^). *R*_s_ is expressed in Ω sq^−1^, which shows its invariability and versatility to a square surface of any size.

At the dawn of the TCF development, Fraser and Cook suggested defining a figure of merit as a simple relationship between the sheet resistance and transmittance of the material:^[^ ^69^ ^]^

(3) $$\mathrm{Fo}M_{1}=\frac{T}{R_{s}}$$

However, Haacke^[^ ^70^ ^]^ showed that this FoM_1_ depends on the film thickness and is higher for nontransparent materials with the maximum value at *T* = 37%. To compensate for the strong weight of the transmittance, Haacke introduced the tenth degree as:

(4) $$\mathrm{Fo}M_{2}=\frac{T^{10}}{R_{s}}=\sigma_{\mathrm{dc}}te^{-10\alpha t}$$

so that the maximal values of FoM_2_ were obtained at a 90% transmittance. Formula (4) is commonly used for transparent conducting films in photovoltaics.^[^ ^50^, ^71^, ^72^, ^73^, ^74^ ^]^ FoMs calculated according to Equations (3) and (4) are not universal parameters, because the values are not constant for the same material and are affected by the film thickness.

Another way to define FoM leans on the equation originally obtained by Glover and Tinkham in 1957 for the transmission losses through a freestanding TCF:^[^ ^75^ ^]^

(5) $$T=1/{\left|1+\sigma_{\mathrm{opt}}t\frac{Z_{0}}{n+1}\right|}^{2}=1/{\left|1+\frac{\sigma_{\mathrm{opt}}}{\sigma_{\mathrm{dc}}}\frac{Z_{0}}{2R_{s}}\right|}^{2}$$

where *σ*_opt_ is the effective optical conductivity, *Z*_0_ is the impedance of free space (377 Ω),^[^ ^76^ ^]^ and *n* is the refractive index of a substrate. In that work, the DC resistance was monitored continuously during irradiation of lead and tin films to measure their transmittance in terms of complex optical conductivity. FoM is expressed as a ratio of electrical and optical conductivities:^[^ ^21^, ^22^, ^77^ ^]^

(6) $$\mathrm{Fo}M_{3}=\frac{\sigma_{\mathrm{dc}}}{\sigma_{\mathrm{opt}}}=\frac{Z_{0}}{2R_{s}\left(1/\sqrt{T}-1\right)}$$

Later, Gordon proposed to consider the film reflectance, *R*:^[^ ^78^ ^]^

(7) $$\mathrm{Fo}M_{4}=\frac{\sigma_{s}}{A}=-\frac{1}{\ln\left(T+R\right)\cdot R_{s}}$$

where *σ*_s_ is the sheet conductance, inversely proportional to *R*_s_. A similar equation was derived based on the Beer–Lambert law by Kaskela et al.^[^ ^79^ ^]^

We need to consider one practical circumstance: in most spectrophotometers, absorbance (*A*) is defined as a decimal logarithm of transmittance: *A* = −log (*T*). Therefore, an upgraded practical FoM, considering the film reflectance, can be presented as:

(8) $$\mathrm{Fo}M_{5}=\frac{\sigma_{s}}{A}=\frac{1}{AR_{s}}=-\frac{1}{\log\left(T+R\right)\cdot R_{s}}$$

Equation (8) is the thickness‐independent relationship suitable for the comparison of TCFs with different transmittance and conductivity values.

To compare the performance of different TCFs, by applying Equation (8), we can also use an equivalent sheet resistance, i.e., a sheet resistance of a film with a certain transmittance, e.g., *T* = 90%:^[^ ^80^ ^]^

(9) $$R_{90}=\frac{1}{\mathrm{Fo}M_{5}\log\left(10/9\right)}=\frac{A\cdot R_{s}}{\log\left(10/9\right)}$$

The number obtained from Equation (9) can be easily compared with the existing industrial TCF standards. For instance, the equivalent sheet resistance of high quality ITO on a rigid substrate is about *R*_90_ =  10 Ω sq^−1^. For clear visibility, in our review paper, we use Equation (9) for the comparison of different TCFs based on SWCNTs and for the estimation of their fundamental limitations.

## 3 Key Principles of SWCNT‐Based TCF Fabrication

**Figure 1:**
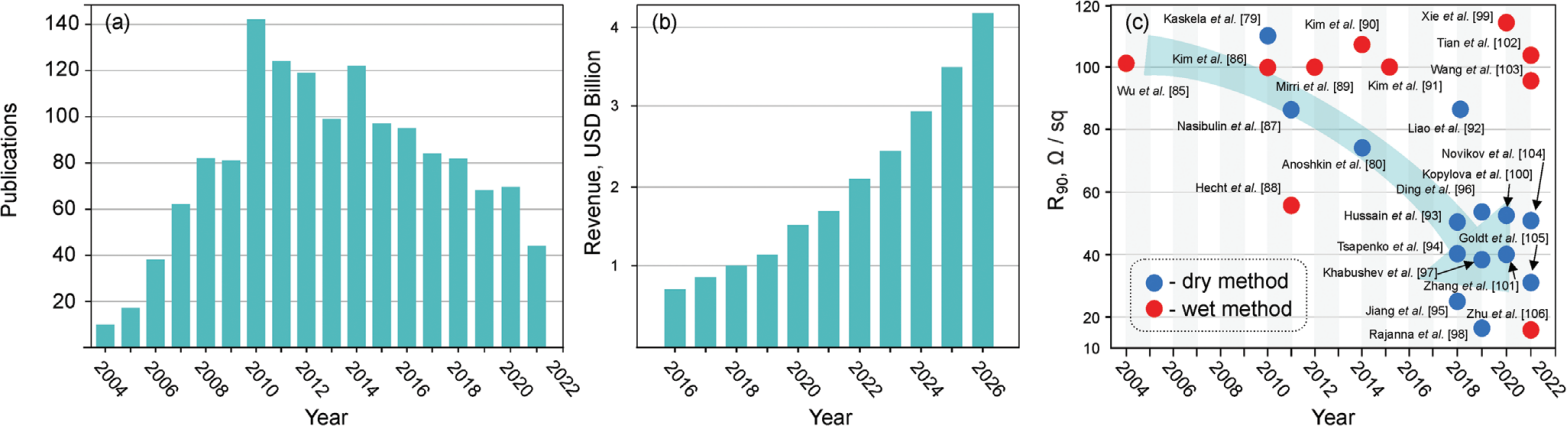
a) Statistics of scientific publications found with keyword combinations: “transparent film” and “carbon nanotubes”; output from Scopus; b) global SWCNT market growth: statistics and forecast (Carbon Nanotubes Market – Global Industry Analysis, 10 May 2021^[^ ^84^ ^]^), c) equivalent sheet resistance values of the SWCNT‐based TCFs (reported for *R_90_* < 120 Ω sq^−1^) fabricated by dry and wet methods.

SWCNT‐based transparent conductive films are one of the most promising candidates to replace traditional TCFs. SWCNTs exhibit ballistic electrical conductivity, superior chemical stability, and high strength and mechanical flexibility.^[^ ^81^ ^]^ TCFs based on SWCNT films possess excellent optical properties: with no haze, low reflectivity, and high contrast for true colors, contrary to metal‐oxide materials. The trend in the number of publications since 2004 (**Figure** **1**a) shows that the SWCNT‐based conducting film research area is well developed and follows the technological hype cycle.^[^ ^82^ ^]^ After the peak of inflated expectations in 2010, we observe a gradual decline in the number of research papers devoted to the SWCNT transparent conductors (Figure 1a). This can be explained by the simultaneous technology‐to‐market transfer (Figure 1b), which is confirmed by an increase in the number of companies commercializing SWCNTs.^[^ ^83^ ^]^

Among various successful market stories, one should mention “Canatu Ltd.” (Finland), “Timesnano” (China), and “OCSiAl” (Russia). “Canatu Ltd.” commercializes CNTs for a wide range of transparent conductor applications from the automobile industry (touch sensors and window heaters) to pellicle technology used in extreme ultraviolet lithography. “Timesnano” is one of the major manufacturers of carbon nanomaterials, including SWCNT‐based commercial powders, dispersions, and pastes for the fabrication. “OCSiAl” is a leader in the production of SWCNT powder that has been demonstrated to form high‐quality TCFs by the wet method. And from now and then, many other industrial attempts appear in the market to apply the unique properties of CNTs as a conductive material: CHASM, Cnano, LG, Arkema, and many others.

Challenges to get high electrical conductivity and stability of SWCNT‐based TCFs superior to other transparent conducting materials remain. Most advanced papers, describing the performance of SWCNT‐based transparent conductors with the equivalent resistance of less than 120 Ω sq^−1^, are gathered in Figure 1c and summarized in **Table** **1**, specifying the fabrication method, type of the substrate, and particular application of the films examined.

**Table 1:** Historical evolution of the SWCNT‐based TCF performance, processes involved to create transparent conductors, and examined applications

Year	Process	Applications	Substrate	*R*_s_ [Ω sq^−1^]	*T* [%]	*R*_90_ [Ω sq^−1^]	Ref.
2004	wet, vacuum filtering	FET	quartz	30	70	102	[85]
2010	dry, HNO_3_ post‐treatment	touch sensor, FET, transparent electrode for OLED	quartz	110	90	110	[79]
2010	wet, spray coating, AuCl_3_ post‐treatment	transparent conductor	quartz	100	90	100	[86]
2011	dry, NO_2_ post‐treatment	air filter, chemical sensor, laser absorber, gas flowmeter, heater, thermoacoustic loudspeaker	free‐standing	84	90	84	[87]
2011	wet, vacuum filtering, chlorosulfonic acid dispersion	transparent conductor	PET	54	90.9	54	[88]
2012	wet, dip coating, chlorosulfonic acid dispersion	transparent conductor	quartz	100	90	100	[89]
2014	wet, spin coating, hybrid with graphene	FET	PET	300	96.4	104	[90]
2014	dry, AuCl_3_ post‐treatment	transparent conductor	quartz	73	90	73	[80]
2015	wet, spray coating, AuCl_3_ post‐treatment	FET	PET	100	90	100	[91]
2018	dry, AuCl_3_ post‐treatment	transparent conductor	quartz	86	90	86	[92]
2018	dry, HNO_3_ post‐treatment	transparent conductor	quartz	51	90	51	[93]
2018	dry, HAuCl_4_ post‐treatment	transparent conductor	quartz	40	90	42	[94]
2018	dry, HNO_3_ post‐treatment	transparent conductor for OLED, FET	quartz	25	90	25	[95]
2019	dry, HNO_3_ post‐treatment	transparent conductor	quartz, PET	57	90	57	[96]
2019	dry, HAuCl_4_ post‐treatment	transparent conductor	quartz	39	90	39	[97]
2019	dry, SWCNT‐ HAuCl_4_‐MoO_3_‐PEDOT:PSS‐carbon fibers heterostructure	transparent electrode for solar cell	quartz	17	90	17	[98]
2020	wet, dispersion in PSS solution	transparent conductor	quartz, PET	115	90	115	[99]
2020	dry, ionic liquid treatment	transparent conductor	quartz	53	90	53	[100]
2020	dry, HNO_3_ post‐treatment	transparent conductor	PET	40	90	40	[101]
2021	wet, spray coating, HNO_3_ post‐treatment, hybrid with rGO	transparent conductor	PET	59	83	104	[102]
2021	wet, spray coating, modification with gallic acid, HAuCl_4_ post‐treatment, hybrid with exfoliated graphite	transparent electrode for OLED	PET	46	80	97	[103]
2021	dry, HAuCl_4_ post‐treatment	transparent conductor	quartz	51	90	51	[104]
2021	dry, HAuCl_4_ post‐treatment (bilateral)	transparent conductor	quartz	9	70	31	[105]
2021	wet, spray coating, modification with tannic acid, hybrid with Ag nanowires and PEDOT:PSS	transparent electrode for OLED	PET	9	83	16	[106]

FET: field‐effect transistor, OLED: organic light‐emitting diode.

### 3.1 Wet and Dry Methods of SWCNT‐Based TCFs Fabrication

**Figure 2:**
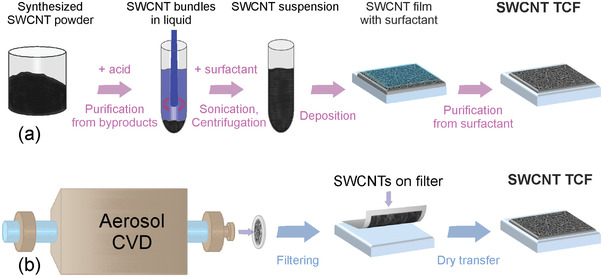
A step‐by‐step description of a) wet and b) dry processes for the SWCNT‐based TCF fabrication.

As described in Table 1, all the techniques for fabrication of SWCNT‐based TCFs can be classified into two groups: wet and dry methods, depending on the state of SWCNT surrounding media (**Figure** **2**).

The wet approach is based on SWCNTs collected as a powder, which first needs to be purified from amorphous carbon and other impurities (Figure 2a). Then the SWCNTs are dispersed by ultrasound treatment in the surfactant solution, which is then followed by centrifugation to remove large aggregates. After the formation of stable dispersion, SWCNTs are deposited onto a substrate applying one of the traditional techniques: vacuum filtration, spray/spin/dip, Langmuir–Blodgett, or Mayer rod coatings. To improve the optoelectronic performance of the SWCNT films, it is important to create a percolated SWCNT network by removing the surfactant from and between the SWCNTs.

The wet approach for the carbon nanotube‐based TCF production was first demonstrated by Wu et al. in 2004.^[^ ^85^ ^]^ This work described the technique based on a nanotube suspension filtration, followed by surfactant elimination and filter membrane dissolving. The resulting thin films exhibited the sheet resistance of 30 Ω sq^−1^ at 70% transmittance (*R*_90_ = 101 Ω sq^−1^). Several publications using the wet fabrication approach appeared over the following three years.^[^ ^107^, ^108^, ^109^, ^110^, ^111^, ^112^, ^113^, ^114^, ^115^ ^]^ For instance, Geng et al.^[^ ^116^ ^]^ achieved *R*_s_ = 40 Ω sq^−1^ at *T* = 70% (*R*_90_ = 135 Ω sq^−1^) by applying a spray technique and subsequent treatment of the obtained film with HNO_3_. This method, as any other using nitric acid doping, faced a sample stability problem, however, demonstrated the optoelectronic performance among the best reported for the wet TCF fabrication approach until 2009.^[^ ^117^, ^118^, ^119^, ^120^, ^121^, ^122^, ^123^, ^124^, ^125^, ^126^ ^]^ Thus, several factors including the source of SWCNTs, the degree and stability of SWCNT dispersions, coating and drying parameters, and the efficiency of removal of the surfactants affect the optoelectronic properties and performance of SWCNT‐based TCFs prepared by the wet process.^[^ ^64^ ^]^

**Figure 3:**
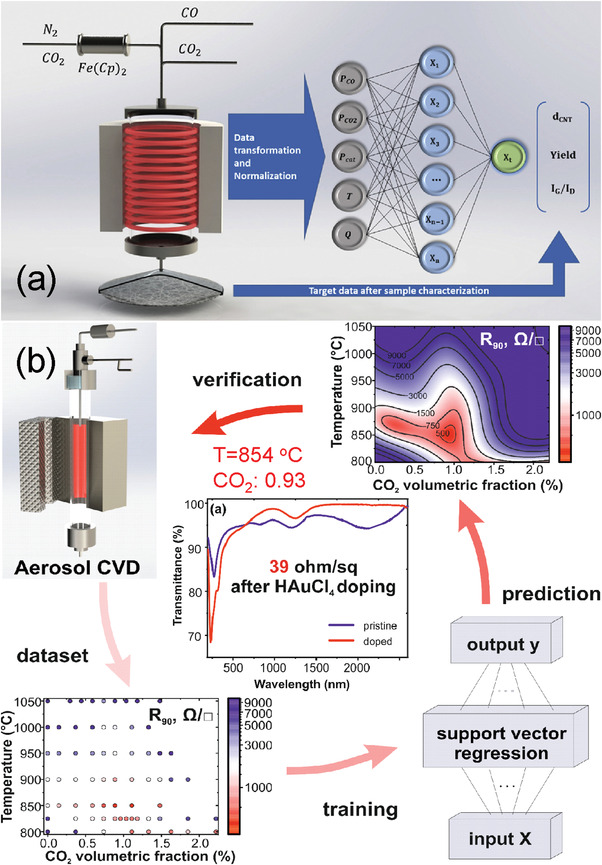
Optimization of SWCNT parameters during synthesis by different machine learning strategies: a) artificial neural network. Reproduced with permission.^[^ ^132^ ^]^ Copyright 2019, Elsevier. b) Support vector regression. Reproduced with permission.^[^ ^97^ ^]^ Copyright 2019, American Chemical Society.

The SWCNT‐based transparent conductors fabricated by the dry method (Figure 1b) involve fewer steps and chemicals. The dry approach (Figure 2b), which is based on an aerosol (floating catalyst) chemical vapor deposition (CVD) method, does not require the use of liquid media. SWCNTs are formed on catalyst particles suspended in the gas phase and usually collected directly downstream of the reactor on a filter as a self‐assembled film of randomly oriented SWCNTs. As the method has fewer technological steps, the resulting SWCNT films contain fewer impurities when compared to the wet technique. However, some synthesis byproducts (like amorphous carbon and catalyst particles) practically always appear in films, therefore stimulating the research to purify the samples, for example, by laser treatment and resistive heating of the freestanding SWCNT films.^[^ ^127^, ^128^ ^]^ For the first time, conducting films fabricated using the aerosol CVD synthesis^[^ ^129^ ^]^ was demonstrated in 2008.^[^ ^126^ ^]^ The films of SWCNTs were transferred from the filter onto a polyethylene substrate by thermocompression, which helped to adhere even short‐length SWCNTs. The first TCFs with promising optoelectrical properties were fabricated in 2010.^[^ ^79^ ^]^ The authors showed the technique for tuning optoelectronic properties of thin SWCNT films by varying bundle lengths during the growth process and achieved the equivalent resistance value of *R*_90_ = 110 Ω sq^−1^. The dry method allowed to fabricate the SWCNT networks suitable for capacitive touch sensors, field‐effect transistors (FETs), and organic light‐emitting diodes (OLEDs). In 2014, an introduction of two different carbon sources in the reactor resulted in the increase of the SWCNT length and therefore a decrease in the equivalent sheet resistance to *R*_90_ = 73 Ω sq^−1^.^[^ ^80^ ^]^ Another improvement was achieved following the proposed strategy^[^ ^130^ ^]^ to affect the Boudouard reaction by adding CO_2_ during synthesis to fine‐tune the diameter of nanotubes (**Figure** **3**b).^[^ ^92^ ^]^ In 2019, Ding et al.^[^ ^96^ ^]^ synthesized SWCNTs from toluene as a feedstock with promising optoelectronic properties (*R*_90_ = 57 Ω sq^−1^). Novikov et al. used a synthetic approach to tune the optoelectronic properties of SWCNT‐based films.^[^ ^104^ ^]^ By increasing the residence time, the authors observed a threefold decrease in *R*_90_ and achieved 51 Ω sq^−1^ after doping by HAuCl_4_.

The conductivity of TCFs strongly depends on the properties of synthesized SWCNTs, namely, diameter, length, presence of defects, and byproducts on the surface.^[^ ^92^, ^131^ ^]^ To affect these parameters and achieve optimum properties, Iakovlev et al. developed an artificial neural network algorithm (Figure 3a),^[^ ^132^ ^]^ which allowed predicting the output characteristics of nanotubes such as diameter, yield, and defectiveness, i.e., Raman *I*_G_/*I*_D_ ratio,^[^ ^133^ ^]^ based on experimental conditions. In the same year, Khabushev et al.^[^ ^97^ ^]^ implemented the support vector regression method to optimize the output parameters of the synthesized SWCNTs and to fabricate the most effective TCF with *R*_90_ = 39 Ω sq^−1^ (Figure 3b).

### 3.2 Postsynthesis Treatment of SWCNT‐Based TCFs

The optoelectronic performance of the SWCNT‐based TCFs is mainly determined by the quality of the produced nanotubes.^[^ ^99^ ^]^ Usually, the as‐deposited SWCNT films do not exhibit *R*_90_ values below 300 Ω sq^−1^.^[^ ^80^, ^94^, ^134^, ^135^ ^]^ Therefore, to enhance the optoelectronic characteristics, the films should undergo some post‐treatment procedures to increase the film conductivity or transmittance.

Adsorption doping is one of the most powerful methods to enhance the efficiency of transparent electrodes. Since every single atom in SWCNTs is on the surface, any impurity on an SWCNT affects its electronic structure, namely, the concentration of charge carriers and position of the Fermi level. The letter affects the Schottky barrier between SWCNTs with different chiralities and metallicities, which is one of the most important factors limiting the total conductivity in the nanotube network. Another type of doping, which can be realized using the substitution of carbon with other atoms like nitrogen^[^ ^136^, ^137^, ^138^, ^139^, ^140^ ^]^ or boron,^[^ ^141^, ^142^, ^143^, ^144^, ^145^ ^]^ modifies electronic structure, however notably induces lattice defects, leading to significant degradation of the charge transfer properties, and is out of consideration in our review.

Effective adsorption doping is a balance between the dopant amount on the SWCNT surface and the resulting film conductivity and transmittance. Usually, a high doping level leads to conductivity improvement, however deteriorating the transparency of the film. Therefore, to reduce the negative transmittance effect, the uniformity and thickness of the dopant layer can be controlled by various film deposition techniques, such as dip‐coating, spin coating, and aerosol deposition. Depending on the doping technique besides the dopant concentration, solvent evaporation and wettability might play a crucial role.^[^ ^94^ ^]^ Interestingly, improvement of the optoelectrical performance (the ratio between initial and resultant equivalent sheet resistances) depends on the quality of nanotubes: the highest factor of improvement is usually observed for lower quality SWCNT films.

Depending on the interaction between dopant molecules and SWCNTs, doping can be divided into n‐type (electron donation from a dopant to a nanotube) and p‐type (electron acceptance). Among n‐type dopants, the first to mention are metals (Li, K, Cu, Zn, Fe, Co, Ni, Ag, Ti, Zr, Eu, Gd, etc.),^[^ ^146^, ^147^, ^148^, ^149^ ^]^ which can be deposited on top or encapsulated inside SWCNTs to be protected from the environment atmosphere. A range of electron‐donating molecules can be precipitated on SWCNTs by simple solution‐based methods and includes dihydro‐nicotinamide adenine dinucleotide (NADH), viologens, polyethyleneimine (PEI), hydrazine, decamethylcobaltocene (DMC), and others.^[^ ^150^, ^151^, ^152^, ^153^ ^]^ The produced n‐type doped SWCNTs rarely exhibit promising optoelectronic properties suitable for TCF applications. SWCNTs at ambient conditions inevitably become p‐doped due to the environmental presence of oxygen and other electron‐withdrawing contaminants. This leads to lowering the Fermi level in the starting material, which reduces the efficiency of electron donation after n‐doping because of the presence of electron acceptor molecules on the SWCNT surface.

For the same reason, p‐type doping is more efficient and stable under ambient conditions. The typical p‐type dopants are acids, Au compounds, SOCl_2_, SOBr_2_, and NO_2_.^[^ ^66^, ^154^ ^]^ Wu et al.^[^ ^85^ ^]^ were the first to obtain highly effective TCFs by HNO_3_ treatment for the wet method TCF fabrication (*R*_90_ = 102 Ω sq^−1^), while Kaskela et al.^[^ ^79^ ^]^ used concentrated nitric acid to dope SWCNT films fabricated by the dry technique (*R*_90_ = 110 Ω sq^−1^). In 2010, Kim et al. were the first who achieved *R*_90_ = 100 Ω sq^−1^ for SWCNT films by doping with AuCl_3_ solution in nitromethane.^[^ ^86^ ^]^ The dopant molecules could be removed by immersion in various solvents, showing their reversibility and providing a way to control the charge carrier concentration. In 2011, the effective SWCNT doping by NO_2_ and chlorosulfonic acid were correspondingly demonstrated by Nasibulin et al. (*R*_90_ = 84 Ω sq^−1^),^[^ ^87^ ^]^ who produced multifunctional freestanding SWCNT films (Table 1), and Hecht et al. (*R*_90_ = 54 Ω sq^−1^).^[^ ^88^ ^]^ Next year dispersion and subsequent doping of SWCNTs in chlorosulfonic acid were proposed by Mirri et al.^[^ ^89^ ^]^ The technique, based on dipping the glass substrate in the SWCNT dispersion, resulted in the fabrication of TCFs with *R*_90_ ≈ 100 Ω sq^−1^. During the following decade, HNO_3_ and AuCl_3_ were the most used and promising dopants for achieving the highest efficiency, as shown in Table 1. In 2018, Jiang et al. achieved the value of *R*_90_ = 25 Ω sq^−1^.^[^ ^95^ ^]^ However, as previously mentioned, the HNO_3_ doping effect is highly unstable and therefore not reproducible because of high volatility of the acid molecules, which cannot be practically implemented for industrial fabrication of TCFs.^[^ ^155^, ^156^, ^157^ ^]^

**Figure 4:**
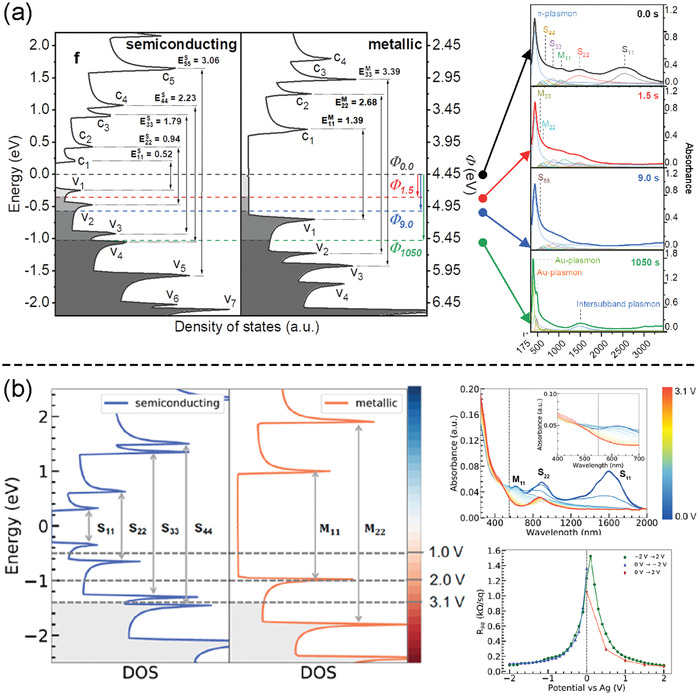
a) Fermi level shift by aerosol doping at different treatment times. Reproduced with permission.^[^ ^159^ ^]^ Copyright 2019, American Chemical Society. b) Fermi level shift attributed to the voltage applied to the SWCNT electrode. Reproduced with permission.^[^ ^100^ ^]^ Copyright 2020, Elsevier. In both cases, a new intersubband peak appears at a high level of doping.

Optimization of SWCNT dimensions during the synthesis allowed to achieve the equivalent sheet resistance of 73 Ω sq^−1^ by drop‐casting of gold chloride solution.^[^ ^80^ ^]^ Later, this result was improved by playing with various solvents (*R*_90_ = 42 Ω sq^−1^)^[^ ^94^ ^]^ and by using the optimized dip‐coating method (*R*_90_ = 36 Ω sq^−1^).^[^ ^158^ ^]^ To control the dopant uniformity on the film surface, Tsapenko et al. proposed aerosol doping, which additionally allowed to fine‐tune the work function of the SWCNT films (**Figure** **4**a).^[^ ^159^ ^]^ That work also confirmed that the high‐level doping of SWCNTs revealed an appearance of an additional peak in the optical spectrum, which corresponded to intersubband plasmon.^[^ ^160^ ^]^ Here, we should also mention a paper by Zhang et al. described an approach of blown aerosol CVD carbon nanotubes: extrusion‐like production of continuous films with a high optoelectronic performance of *R*_90_ = 40 Ω sq^−1^.^[^ ^101^ ^]^ The possibility of bilateral doping of SWCNTs has been recently demonstrated by Goldt et al.^[^ ^105^ ^]^ It was shown that nanotube opening led to the HAuCl_4_ penetration inside SWCNTs, in addition to the dopant presence on the surface, and efficient advancement of the optoelectrical properties (*R*_90_ = 31 Ω sq^−1^).

Kopylova et al. demonstrated a reversible and efficient electrochemical n‐ and p‐doping of SWCNTs (*R*_90_ = 53 Ω sq^−1^) using an ionic liquid.^[^ ^100^ ^]^ The Fermi level in the SWCNTs was controlled by applying a certain potential to SWCNTs in a three‐electrode cell, resulting in the Fermi level shift up or down for over 1.0 eV (Figure 4b).

Thus, we have briefly described the techniques for fabrication of SWCNT‐based TCFs, deliberated approaches to enhance and control the doping level in the SWCNTs, and discussed the achieved state of modern SWCNT‐based TCFs. Further, we will focus on the potential strategies to boost the optoelectronic performance and set the fundamental limit for the transparent films based on carbon nanotubes.

## 4 Rational Design of TCFs and Fundamental Limit

Let us discuss some examples of the rational design of TCFs, which might enhance the optoelectronic performance of SWCNT films. The rational design of TCFs can be considered as an approach to creating a composite TCF that exhibits better properties than its constituents or as a design of certain conducting geometry providing high conductivity and transmittance.

For the first time, the rational design was used by Zhu et al. to fabricate TCFs based on a hybrid comprising the metallic grid and graphene.^[^ ^161^ ^]^ They achieved the sheet resistance of 3 Ω sq^−1^ at the transmittance of 80% (*R*_90_ = 20 Ω sq^−1^). The rational design applied for graphene and carbon nanotubes by Kim et al. allowed to fabricate extremely transparent composite films.^[^ ^90^ ^]^ The hybrid material was applied as a FET with the on‐state current of 0.2 mA outperforming pristine graphene (0.1 mA) and SWCNTs (0.028 mA). The authors decreased the equivalent sheet resistance from 104 Ω sq^−1^ by a factor of 3, applying AuCl_3_ as a dopant.^[^ ^91^ ^]^ Later, the combination of graphene (or reduced graphene oxide) and SWCNTs has been applied by several groups.^[^ ^102^, ^103^, ^162^ ^]^ Zhu et al.^[^ ^106^ ^]^ revealed a method for producing a three‐layer film based on SWCNTs modified by tannic acid, Ag nanowires, and PEDOT:PSS with extremely good optoelectrical properties: *R*_90_ = 16 Ω sq^−1^. Similar results were demonstrated by Rajanna et al. in 2020 (*R*_90_ = 17 Ω sq^−1^) for SWCNT‐based TCFs, which were successfully implemented in amorphous Si solar cells.^[^ ^98^ ^]^ The rational design was achieved by the coating of SWCNTs with MoO_3_ (dopant with high work function^[^ ^163^ ^]^), followed by doping with HAuCl_4_ and PEDOT:PSS deposition. As the last step, SWCNT fibers, obtained by the wet pulling method,^[^ ^164^ ^]^ were deposited as current collectors to increase the conductivity of the electrode. This direction of the rational design eventually resulted in complex multilayer hybrid materials based on carbon nanotubes.

**Figure 5:**
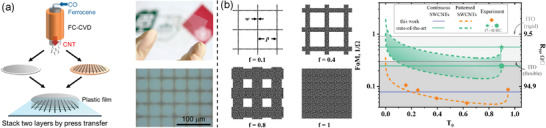
Patterned SWCNT TCFs. a) Fabrication process of the SWCNT TCF with a microgrid used during the SWCNT collection. Reproduced with permission.^[^ ^166^ ^]^ Copyright 2014, American Chemical Society. b) Dependence of the optoelectronic performance of patterned films on the initial transmittance of an SWCNT film. Reproduced with permission.^[^ ^165^ ^]^ Copyright 2020, American Chemical Society.

Another approach to the rational design of TCFs can be realized by film patterning, i.e., by creating narrow, nontransparent, but highly conductive paths at overall high film transmittance. This strategy might help overcome the conductivity–transmittance trade‐off for the SWCNT‐based TCFs.^[^ ^165^ ^]^ The patterning might be realized either on a filter during the SWCNT collection or afterward from the collected continuous film. Ohno et al.^[^ ^166^ ^]^ created micropatterns (with a period of 37.5 µm) on a filter when collected aerosol‐synthesized SWCNTs at the reactor outlet (**Figure** **5**a). The lithography technique used to the pattern fabrication allowed to reduce the equivalent sheet resistance from 201 to 112 Ω sq^−1^.

The improvement of optoelectronic properties of patterned films was thoroughly examined by Mitin et al.^[^ ^165^ ^]^ Using a combination of optical laser lithography and dry‐etching in oxygen plasma, they demonstrated that patterning of continuous SWCNT layers might increase the overall transmittance, *T*, without losses in conductivity. The authors have derived the dependence of *T* on *R*_s_ for the patterned films [Equation (10)]:

(10) $$T=1-f+f\exp\left(-\frac{\alpha \rho }{R_{s}(1-\sqrt{1-f}}\right),$$

where *ρ* is the resistivity of carbon nanotube films, *f* is the filling factor of a grid, which is equal to 1 for a continuous film and tends to 0 for highly perforated films (Figure 5b). The authors correlated initial film transmittance (*T*_0_) with the resulting sheet resistance of the patterned film:

(11)) $$R_{s}=\frac{-\alpha \rho /\ln T_{0}}{1-\sqrt{1-\frac{1-T}{1-T_{0}}}}$$

To achieve the equivalent sheet resistance below ITO by patterning, one should select a film with *T*_0_ < 10%. Other aspects and general patterning approaches are described in a full‐scale review paper.^[^ ^167^ ^]^

**Figure 6:**
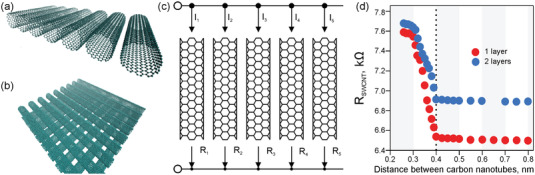
Ideal SWCNT‐based TCFs: a) one‐layer and b) two‐layer films based on armchair SWCNTs; c) equivalent circuit of the film of aligned SWCNTs; d) resistance of one SWCNT in the equivalent circuit as a function of the distance between SWCNTs.

Let us elaborate on an ideal SWCNT network with the highest optoelectronic performance to set the fundamental limit of the TCFs based on SWCNTs. To formulate the concept, we should construct a conductive network without hopping and scattering of charge carriers. For that purpose, let us consider a film comprising parallel defect‐free SWCNTs (**Figure** **6**a). Such kind of network provides conductivity only in one direction. To fabricate a non‐unidirectional conducting surface, the film should contain at least two layers and, in the simplest case, positioned at a 90° angle between each other (Figure 6b). These two configurations provide the best TCF performance for unidirectional and bidirectional networks, assuming that the adequate components to build the network are selected. As the conductivity of double‐walled and multiwalled CNTs is provided by their outer layers, SWCNTs outperform those nanotubes because of higher optical transmittance.^[^ ^168^ ^]^

We calculated the electrical conductivity of individual SWCNTs in terms of the transmission coefficient according to Landauer–Buttiker formalism employing Keldysh nonequilibrium Green function technique and original method for transmitance calculations.^[^ ^171^ ^]^ Among all configurations, only armchair SWCNTs possess purely metallic (zero bandgap) properties, while the others are classified as semiconducting and semimetallic types.^[^ ^169^ ^]^ Therefore, we used ballistically conductive armchair SWCNTs (with the quantum resistance of 6.46 kΩ)^[^ ^170^ ^]^ and constructed a film of aligned SWCNTs as parallel‐connected resistors (Figure 6c). Here, the resistance of a film can be estimated as:

(12) $$\frac{1}{R_{s}}=\sum {\frac{1}{R}}_{i}$$

(13) $$R_{s}=R_{\mathrm{SWCNT}}/n$$

where *n* is the number of SWCNTs in a film, and *R*_SWCNT_ is a resistance of a metallic SWCNT. Figure 6d shows the dependence of the SWCNT resistance on the gap between tubes. If the distance is too small, the overlap of *π* electronic clouds increases the resistance. The interaction of electronic clouds disappears, and the resistance decreases to the quantum resistance value for an SWCNT in the monolayer film, when the distance between nanotubes exceeds 4 Å. The resistance here does not depend on the diameter of SWCNTs. For the double‐layered TCF, the minimal resistance is higher (6.84 kΩ) because of the contact between layers and the scattering of charge carriers. As we consider the ideal defect‐free model, these resistance values are constant in frames of ballistic transport for any length of nanotubes.

**Figure 7:**
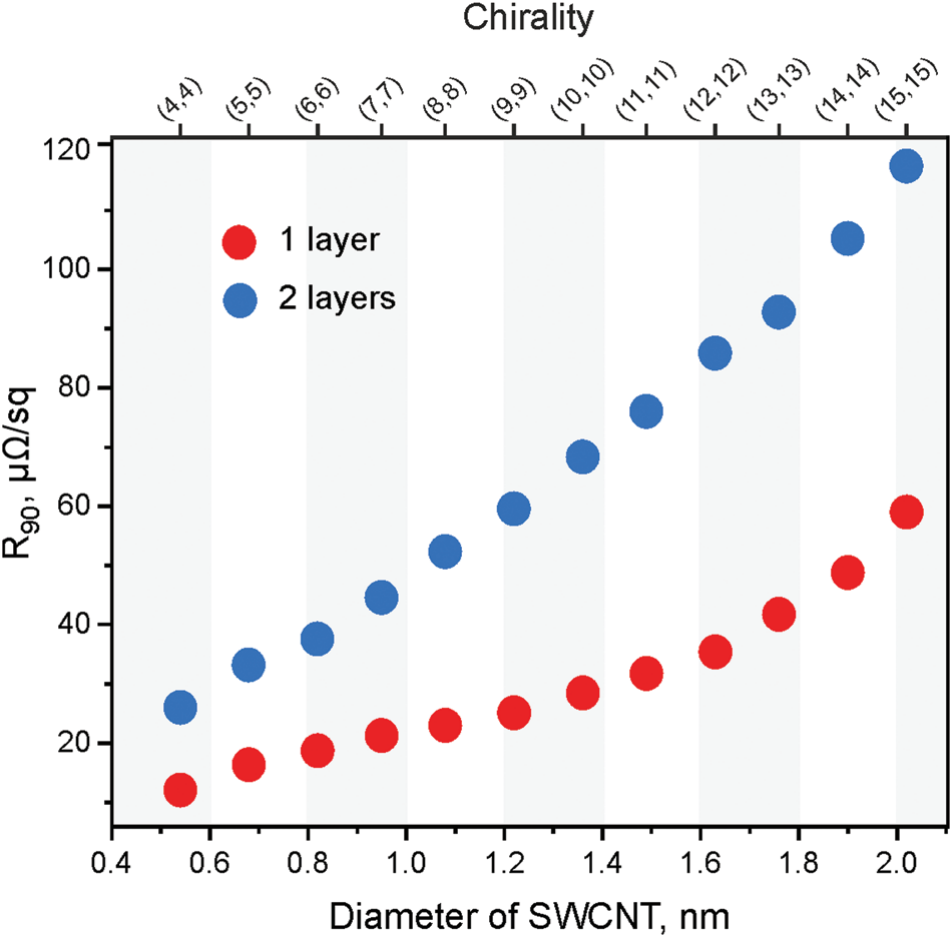
Dependence of *R*_90_ on chirality/diameter of SWCNTs in case of ideal films of ordered armchair SWCNTs.

As we emulate the scale of real‐size devices using TCFs, we carried out the *R*_90_ calculations for the films with dimensions of 10 × 10 cm^2^. Here, we estimated the number of nanotubes that can be placed in 10 cm length at a distance of 4 Å between each other. The results of the calculations according to Equation (13) and considering the absorbance at a 550 nm wavelength for one‐ and two‐layer films are shown in **Table** **2** and **Figure** **7**. Obviously, the smaller the diameter of the SWCNTs the more nanotubes could be placed to create a TCF. The films comprising (4,4) SWCNTs possess the best possible characteristics for TCFs: *R*_90_ = 14 and 22 µΩ sq^−1^ for 1D‐ and 2D‐conducting films, respectively. These values are the fundamental limits for the optoelectronic performance of SWCNT‐based films.

**Table 2:** Sheet resistance and transmittance for one‐ and two‐layer films (Figure 6a,b, respectively) based on ordered ideal armchair SWCNTs at a distance of 4 Å between each other

Chirality	*D*_CNT_ [nm]	*R*_S_ 1 layer [µΩ sq^−1^]	*T* (550 nm), % 1 layer	*R*_S_ 2 layers,[µΩ sq^−1^]	*T* (550 nm), % 2 layers	*R*_90_ 1 layer,[µΩ sq^−1^]	*R*_90_ 2 layers [µΩ sq^−1^]
(4,4)	0.54	61	97.6	64	96.5	14	22
(5,5)	0.68	70	97.6	74	95.5	16	32
(6,6)	0.82	79	97.5	83	95.4	19	37
(7,7)	0.95	87	97.5	92	95.2	21	43
(8,8)	1.08	96	97.5	101	94.7	23	52
(9,9)	1.22	105	97.5	111	94.5	25	59
(10,10)	1.36	114	97.4	120	94.2	28	68
(11,11)	1.49	122	97.3	129	94.0	32	76
(12,12)	1.63	131	97.2	139	93.7	35	86
(13,13)	1.76	140	96.9	148	93.6	42	93
(14,14)	1.90	149	96.6	157	93.2	49	105
(15,15)	2.02	156	96.1	166	92.8	59	117

*D*_CNT_: diameter of SWCNTs of the corresponding chirality that constitute the film; *R*_s_, *T* (550 nm), and *R*_90_: sheet resistance, transmittance at a wavelength of irradiation 550 nm, and equivalent sheet resistance of the film, respectively.

Thus, transparent electrodes based on carbon nanotubes have a giant potential to be improved and outperform all known materials: ITO and metal films. Therefore, this work reveals the fundamental limit for TCFs based on SWCNTs and opens new and encouraging horizons for the development of this field.

## 5 Beyond Existing Materials: Stretchable SWCNT‐Based TCFs

SWCNT networks can be applied to fabricate not only flexible but also stretchable devices, opening new horizons in optoelectronics beyond the currently used rigid and brittle transparent conductors. Thin SWCNT films exhibit crack‐free elasticity and optical transparency, so they provide additional advantages for the applications as electrodes for wearable electronics.^[^ ^172^ ^]^ Several functional stretchable electronic devices based on carbon nanotubes have been already demonstrated, including active strain gauge,^[^ ^18^, ^172^, ^173^ ^]^ pressure sensor,^[^ ^174^ ^]^ OLEDs,^[^ ^175^ ^]^ skin‐like passive electrode,^[^ ^18^ ^]^ and supercapacitor.^[^ ^176^, ^177^, ^178^ ^]^

**Figure 8:**
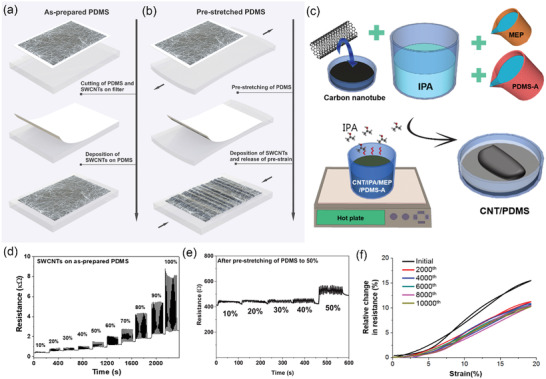
Generic illustration of different approaches to fabricate SWCNT‐based TCFs and corresponding strain tests with measured relative resistance change: a,d) SWCNTs deposited onto the as‐prepared substrate. Reproduced with permission.^[^ ^179^ ^]^ Copyright 2019, American Chemical Society. b,e) SWCNTs deposited onto the prestretched substrate. Reproduced with permission.^[^ ^179^ ^]^ Copyright 2019, American Chemical Society. c,f) SWCNTs embedded into elastomer composite structures. Reproduced with permission.^[^ ^188^ ^]^ Copyright 2018, Springer Nature.

Fabrication of stretchable conducting films could be realized either by transferring SWCNT films onto nonstretched (**Figure** **8**a) or prestretched (Figure 8b) elastomeric substrates,^[^ ^179^ ^]^ so that the same film of SWCNTs depending on the way it is deposited possess oxymoron properties. SWCNTs can be very sensitive to stretching (Figure 8d) or vice versa, they can be used as electrically stable under stretching films (Figure 8e). Gilshteyn et al.^[^ ^179^ ^]^ fabricated highly stretchable and transparent electrodes by these two approaches and compared their performances. The sheet resistance of the samples on the as‐prepared PDMS increases about three times after stretching: from 100 Ω sq^−1^ at 0% strain (*R*_90_ = 31 Ω sq^−1^) to 320 Ω sq^−1^ at 50% strain (Figure 8d). In contrast, in frames of the prestretching approach (to 50%), the resistance of a structure does not practically change at 10%–40% strains and responded to the mechanical influence only at the pre‐stretched value of 50% (Figure 8e).

The first approach results in the alignment of randomly oriented SWCNT networks while stretching. These unidirectional, transparent, and stretchable CNT sheets were used by Yamada et al.^[^ ^173^ ^]^ for the fabrication of the SWCNT strain sensor with high durability (10 000 cycles at 150% strain). Transparent SWCNT films deposited on PDMS by Zhou et al. as shown showed an initial sheet resistance of 170 Ω sq^−1^ at 87% optical transmittance (*R*_90_ = 42 Ω sq^−1^), which became about 2.5 times the initial resistance under 100% strain.^[^ ^180^ ^]^ Experimental and computational results of correlation between applied strain, microstructural evolution, and electro‐optical properties allow to understand and underline mechanisms for the building blocks for stretchable electronics based on SWCNT films. Jin et al.^[^ ^181^ ^]^ showed that the hysteretic resistance evolution is governed by a microstructural parameter, such as the mean projected CNT length ratio over the film length. The effect of strain history on resistance implies that SWCNT films can be “programmed” by the first cycle of strain and release, to be reversibly stretchable within the range defined by the first strain, as investigated by Lipomi et al.^[^ ^174^ ^]^

The second approach results in the formation of so‐called wrinkles when the strain is released.^[^ ^182^, ^183^ ^]^ Under stretching, the wrinkles are smoothed, so that the total length of the conductor does not change, therefore keeping the resistance constant. This strategy of using a prestretched substrate is commonly used for the creation of heaters and supercapacitors since crumpling increases the amount of surface area available in a small amount of space, which increases the amount of charge it can hold.^[^ ^184^, ^185^, ^186^, ^187^ ^]^ Both approaches are applicable for deposition on stretchable and biocompatible hydrogel substrate to create skin‐like passive electrodes and active finger‐mounted joint motion sensors.^[^ ^18^ ^]^

Another method is based on the formation of 3D composite stretchable materials, in which SWCNTs are uniformly dispersed within the elastomeric matrix, for example, PDMS (Figure 8c).^[^ ^188^ ^]^ The nanohybrids demonstrated low sheet resistance values (less than 20 Ω sq^−1^), high elasticity (40% strain), and good strain sensitivity and stability (up to 10 000 stretch/release cycles with a maximum relative resistance change of 20%) as shown in Figure 8f. Porous SWCNT 3D networks produced by PDMS penetration into the SWCNT aerogel are also an example of this method.^[^ ^189^ ^]^ These highly stretchable and transparent (maximum strain is 250% and *T* = 90%) bulk electrodes (30 µm) showed no significant changes in resistance after 20 cycles of 100% stretching. Highly stretchable (>150% strain) and conductive (conductivity > 132 S m^−1^) opaque electrodes were fabricated by welding the junctions of CNTs using graphite nanoplatelets followed by infiltrating with PDMS—3D interconnected conductive networks.^[^ ^190^ ^]^ Unfortunately, the major drawback of the composite‐based approach is the low transparency of the fabricated structures, since usually for conductive percolation network inside elastomer a high concentration of CNTs is required.

Applications of SWCNT networks in thin‐film transistors and integrated circuits, as stretchable TCFs in various sensors and devices, and as stretchable electrode materials owing to new form factors such as flexibility and stretchability are already shown by various research groups.^[^ ^64^, ^180^, ^191^, ^192^, ^193^, ^194^ ^]^ The key points for further research in this area are also to improve optoelectronic performance, deeper understand the microstructural origin of resistance–strain dependence, and integrate such stretchable SWCNT‐based structures along with other compliant rigid interfaces. Developing a stretchable electrode that is transparent as well is especially important for prospective applications in the rapidly growing fields of wearable electronics and Internet of Things, which are shaping the function and view of future electronic devices.

## 6 Conclusions

Over the last 30 years, significant development of techniques for the SWCNT synthesis and thin‐film fabrication resulted in the production of superior SWCNT‐based transparent conductors exhibiting properties close to the ITO performance. Here, we reviewed the main techniques for the fabrication of rigid, flexible, and stretchable SWCNT‐based TCFs, the methods for their evaluations, and emulated the fundamental limit for the SWCNT film performance.

First, we have reviewed various figures of merit for TCFs and proposed to use the approach, which might be convenient for an appropriate comparison of films with different thicknesses. Equivalent sheet resistance, i.e., the sheet resistance of the film with 90% transmittance (at 550 nm), allows a straightforward comparison of the optoelectronic properties of transparent conductors.

Second, we have overviewed the development in the field for the last 18 years with a focus on the best optoelectronic properties of the SWCNT‐based TCF materials obtained on rigid and flexible substrates.

Third, based on the rational design, we have reviewed the approach for the future development of the TCF materials. We constructed the ideal SWCNT network and calculated the best possible performance, i.e., we found theoretical limits for the TCFs based on unidirectional and two‐layered conducting films of SWCNTs, to be correspondingly *R*_90_ = 14 and 22 µΩ sq^−1^. These extraordinarily low values of sheet resistances demonstrate a huge potential for further development of the field of transparent conductors based on SWCNTs.

Finally, we reviewed transparent conductors beyond rigid and flexible materials and discussed various methods for fabricating stretchable 2D electrodes with high sensitivity or stability under stretching conditions and 3D composite materials with SWCNTs well dispersed in an elastomer matrix. The main development directions for further research are discussed. Stretchable and transparent electrodes are important for prospective applications in the rapidly growing fields of wearable electronics and Internet of Things.

## Conflict of Interest

The authors declare no conflict of interest.
